# Meetings of Societies

**Published:** 1903-03

**Authors:** 


### MEETINGS OF SOCIETIES.

### Bristol Medico-Chirurgical Society.

*December 10th, 1902.*

Mr. G. MUNRO SMITH, President, in the Chair.

Dr. WALTER C. SWAYNE showed a **specimen of cystic fibroid of the uterus removed by panhysterectomy.** The patient was a multipara, aged 53. An abdominal tumour had been noticed for two years. Then menorrhagia and, later metrorrhagia, occurred, the latter persisting from May to October of this year. When seen by the speaker the hemorrhage was fairly free and fœtid. As malignant disease was suspected, it was decided to explore and curette the uterine cavity. The uterus was then found to be myomatous. Hemorrhage occurred again in November, but only in small amount. Then panhysterectomy was performed. A gush of fluid followed the introduction of the myoma-screw. On section of the uterus after removal, a large cyst was found in the wall of the uterus on the left side near the fundus, and near this a second one, and in another place a third. Microscopical examination had shown that the tumour was a myoma, and the sections showed a rather curious condition which illustrated the origin of the cysts. Canals were seen with myxomatous cells in their interior. In certain places there were larger spaces, some filled with myxomatous cells, apparently lined with endothelium. Since the operation a slight watery vaginal discharge had been observed which the speaker had reason to believe came from a ureter, probably the result of a small slough.

Mr. T. CARWARDINE showed **a patient whose left lung had been drained.** The patient was a man of 37, who had bronchiectasis and a fibroid condition of the lower lobe of the left lung. At the time of operation he had been very ill, and expectorating a pint of fœtid sputum daily. It was considered that his only chance of recovery was to drain the affected portion of the lung. Three pieces of rib; viz., of the seventh, eighth, and ninth, each about four inches in length, were removed posteriorly. At a second operation it was intended to remove a portion of the lung to drain the bronchial tubes. After cutting into the lung a certain distance a cavity was found containing fœtid pus. The cavity was packed, after arresting hemorrhage with ligatures and the cautery. After the operation the expectoration soon became quite sweet, and now there was no expectoration at all. The patient could blow air through the drainage-track. It was intended to make the opening a permanent one.—Dr. Markham Skerritt remarked that the operation had given better results than were usually obtained in such cases. Physicians had several times diagnosed and localised cavities which surgeons had been unable to find at subsequent operations.

Dr. F. H. Edgeworth read a paper on **some of the initial signs of pleurisy.**—Mr. H. F. Mole mentioned that cases of pleurisy were met with entirely without pyrexia.—Mr. Roger Williams stated that in the form of pleurisy met with in association with pelvic tumours none of the above symptoms were usually found. The relations between the pelvic tumour and the pleurisy were not clear. It was met with in ordinary cases of ovarian cyst, but more commonly in cases of malignant tumours of the pelvic organs. According to the embolic theory the tumours, by compressing the pelvic veins, led to thrombosis and embolism, then set up pleuritic inflammation. Sometimes the pleurisy was due to malignant metastasis. In these cases there were hardly any initial symptoms, the first indication of pleurisy often being the discovery of effusion.—Dr. D. A. Alexander alluded to the possible co-existence of peritonitis and pleurisy, though in other cases the abdominal symptoms were reflex.—Dr. Michell Clarke pointed out that when a pleuritic rub was found together with abdominal pain it was very necessary to bear in mind the possibility of the primary seat of infection being in the abdomen, and of the infection having spread through the diaphragm. Pure diaphragmatic pleurisy seemed to be an extremely rare affection, *i.e.*, when not due to a primary abdominal lesion. With regard to hæmoptysis, he held the view that the majority of the cases with that symptom were tubercular in origin. The absence of the physical signs of a tubercular affection did not exclude its existence, and the cases should be carefully watched. Tuberculin might be injected in doubtful cases to clear up the diagnosis.—Dr. Markham Skerritt said that until recently the view had been taken that nearly all cases of pleurisy were tubercular. He would be very doubtful himself as to the absence of tubercle in a case which began with hæmoptysis. It was rare to find a pure diaphragmatic pleurisy, but the diaphragm might bear the brunt of the attack. He had recently seen a case in which there was great tenderness anteriorly, due to pleuritis existing posteriorly. —Dr. WATSON WILLIAMS alluded to the practice in Germany of giving iodide of potassium in cases in which tuberculosis was suspected. If local manifestations arose then the fact was regarded as evidence of tubercle. The pain in pleurisy was often referred, and he always carefully examined the whole course of the nerves along which the pain was referred. He described a case he had recently seen in which pleurisy with effusion ran its course without any pain. He was inclined to think the less acute cases, those with the less-marked clinical signs, were the most likely to be tubercular.

Dr. J. O. SYMES read a paper on the **presence of diphtheria bacilli in atrophic rhinitis.** In true atrophic rhinitis there was pallor of the mucous membrane, atrophy of bone structures, formation of crusts, cachexia, and leucocytosis. The speaker had bacteriologically examined twenty-three cases. Of these twenty were females. The ages of the patients had varied from 9 to 57 years, the average age being 20 years, and the average duration 7 years. The ozœna was noticeable in all except one. In 87 per cent. of the cases he had found a bacillus present which resembled the Klebs-Loeffler bacillus. In 7 per cent. the bacillus mucosus had been found. A series of healthy noses had been examined in a similar way. In none of these had bacilli like diphtheria-bacilli been found, but in many a short bacillus of the Hoffman type was present. Was the long bacillus found in atrophic rhinitis the true diphtheria bacillus, or one which morphologically and culturally resembled it? The possibilities of error in answering this question were very great. The most satisfactory test was that by inoculating the bacillus into animals. In two of his cases this had been done, and the report stated that the bacilli were those of diphtheria, and virulent. The occurrence of the bacilli in these cases did not prove that they were the cause of the atrophic rhinitis. If they were the cause, then atrophic rhinitis had to be regarded as chronic diphtheria. In conditions simulating atrophic rhinitis; namely, syphilitic rhinitis, rhinitis sicca, etc., diphtheria-like organisms were not found, but the bacillus mucosus was frequently present. One of the speaker's cases of rhinitis, in which the bacillus had been found, had been excluded from school by the health authorities for nine weeks. Prof. Klein had reported the bacillus present to be a virulent form of the diphtheria bacillus. Frequent subsequent examinations had shown that the organism was constantly present. It was there even sixteen months after its first detection. — Dr. D. S. DAVIES alluded to the numerous difficulties diphtheria presented to those responsible for the health of towns. When the bacillus was found in the throats or noses of school children it was difficult to see what other course than that of isolating the children could be taken. In some American cases the organism seemed to have persisted for ten or more years in the nasal sinuses. It was not settled what should be done in such cases. When he found them co-existing with cases of diphtheria he took a middle course with regard to isolation, and prevented the children from attending school.—Dr. WATSON WILLIAMS asked if the child whose case had been mentioned by Dr. Symes had associated with other children, and if so, what had been the result. A great many cases were going about with bacilli in the throat and nose indistinguishable from the diphtheria bacillus. He had tried the treatment of atrophic rhinitis with anti-diphtheritic serum when it was first recommended some six years ago, but had not obtained good results. —Dr. EDGEWORTH asked if there was any evidence that these cases were a source of the dissemination of diphtheria.—Dr. SYMES stated further investigations would be required before it would be possible to state that the organism described was a true diphtheria bacillus.

Mr. J. PAUL BUSH reported a case of **tumour of the stomach,** in which he had removed the growth and one-third of the stomach; and the patient, the specimen, and some lantern illustrations of the operation and of the structure of the growth were shown. The patient was a woman of 61, who had had good health until a year previously, when indigestion and loss of weight developed. Six months previously abdominal pain and vomiting began, and these symptoms had gradually increased. There was no history of hæmatemesis; there were no signs of gastric dilatation. A tumour, the size of the fist, was felt in the lower epigastric region. It moved with respiration. It could not be made out whether the tumour was in the stomach or the colon. At the operation it was found to affect the pylorus and the adjacent third of the stomach. Partial gastrectomy was performed; the duodenum was joined to the stomach end-to-end. The operation took two hours, and the patient was considerably collapsed at the end. On the third evening after the operation the patient had, by mistake, been given some bovril and cocoa. She retained this. Her recovery had been uninterrupted.—Dr. EDGEWORTH remarked that the symptoms, when he had seen the case, seemed rather to suggest that the growth was in the colon. In a similar operation, in which he had given the anæsthetic, he had injected a small quantity of atropine. Such an injection seemed to guard against the cardiac inhibition, which was otherwise liable to develop as a result of handling the stomach.

Prof. E. FAWCETT showed a **series of lantern slides illustrating the histology and development of the central nervous system in the embryo.**

*January 14th, 1903.*

Mr. G. Munro Smith, President, in the Chair.

Mr. Paul Bush showed a specimen of **fibroid of the uterus removed by hysterectomy.** The fibroid, which was a moderately large one, had been impacted in the pelvis, and had caused intestinal obstruction, necessitating its removal. The patient was a woman of 67 years of age. The tumour had been easily detected on examination. Symptoms had only been noticed for a month. In conjunction with the fibroid, a papillary cyst of one ovary had been removed, and was shown in the specimen. For a fortnight after the operation the patient went on well; then pain in the right loin was noticed, and a few days later a large fluctuant swelling formed there. Through a lumbar incision about a quarter of a pint of pus mixed with urine was evacuated from the swelling. On analysis, the fluid which escaped was shown to contain urea, uric acid, phosphates, chlorides, and hippuric acid. The question as to the advisability of performing a further operation had to be considered. To determine whether the urine came from the ureter or bladder, methylene blue was first injected into the bladder and found not to colour the discharges from the wound; then the drug was given by the mouth, and very soon the pus was stained blue. It had been concluded therefore, that the urine came from an injured ureter. The abscess gradually healed up without a further operation.—Dr. W. Hey Groves remarked on the nature of the cyst, and on that of the complication in the after-course of the case. He considered the complication had been a dangerous one.—Mr. J. Lacy Firth agreed with the last speaker. The late formation of the abscess appeared to show that it had resulted from an injury to the ureter, which had led to secondary sloughing and leakage. It was interesting to note the advances which had been made in the methods of treatment of ureteral injury when the injury was discovered during an operation. Until about ten years ago the proper treatment had been considered to be nephrectomy; now attempts would always be made to suture the wound or the division in the ureter, or to engraft the proximal end on the bladder. If so much of the ureter had been accidentally removed that it was impossible to affect the junctions mentioned, even then nephrectomy need not be performed; instead, the ureter might be simply ligatured; the result in the majority of cases would then be atrophy of the kidney, and not the production of hydro-nephrosis.—Dr. Walter Swayne alluded to a somewhat similar case, in which a ureter had been injured; but in his case the leakage of urine had been into the vagina. This still continued in slight degree, though the patient was in all other respects well.—Mr. Bush remarked that the abscess, so far as he could judge, had been completely retro-peritoneal, and thought the leakage of urine had begun about the eighth day.

Dr. H. Edwards Stack showed **a series of contracted pelves**, of various types, obtained from various places. The set he had collected was almost a complete one for teaching purposes, and belonged to the Bristol Royal Infirmary. He considered the best classification of deformed pelves was a clinical one, and suggested that they should be considered in three main groups; viz., firstly, the pelves with slight deformity, and through which a living child might be delivered, though with more or less difficulty; secondly, those which did not permit of the delivery of a viable child, and so rendered craniotomy necessary; and, thirdly, those through which delivery was impossible. Dr. Stack then described the various specimens.—Dr. Walter Swayne complimented the last speaker upon the fine collection of pelves he had exhibited. The classification of contracted pelves, according to their clinical effects, was better than that usually given in the books. The proportion of contracted pelves was very low in Bristol, and the Bristol women seemed very capable of bearing children, and also of bringing up children successfully.

Mr. A. W. Prichard reported a case of **rupture of the intestine**, successfully operated upon. The patient, a man of 22, had been struck in the abdomen by the shaft of a cart when cycling. When first seen by the speaker the patient was not collapsed. A rent in the rectus muscle was felt. Some hours later the patient became restless and vomited, and the abdomen was rigid. On opening the abdomen an irregular rent in the small bowel was found, the peritoneal coat being torn longitudially, the mucous and muscular coats transversely. No bowel contents had escaped. The mesenteric glands were enlarged, and there were tubercles in the peritoneum. Presumably paralysis of the bowel with absence of peristalsis explained the absence of extravasation of bowel contents into the peritoneal cavity.—In answer to Mr. Morton, it was stated that no free gas had been found in the peritoneum.

Mr. H. G. Kyle read notes on **a case of perforated typhoid ulcer for which laparotomy had been performed.** (See p. 30.)—Dr. J. O. Symes stated that the patient's death had not been due to the ulcer, the perforation in which had been successfully closed. He attributed the measure of success secured to the early diagnosis of the perforation by the house-surgeon, Mr. Martin, and to the promptness and rapidity with which the operation had been performed. In the obscurer cases of perforation of the bowel in typhoid fever, he considered that the following symptoms were of the greatest value in diagnosis:—(1) Pain. Without perforation there might be colicky pain, but severe and increasing pain was an important indication of perforation. (2) Profuse sweating. (3) Obliteration of the liver-dulness.—Mr. Paul Bush alluded to a case of perforation in which he had operated. The patient had died a week later from a second perforation. He considered the outlook was very bad after operation in these cases. To excise a large portion of the bowel, he considered would be madness.—Mr. W. H. Harsant had operated twice in such cases. Both patients died. He alluded to a case he had seen in which two other practitioners had urged operation, but he had refrained, feeling a doubt as to the existence of a perforation. The patient got quite well.—Dr. H. E. Stack considered the anæsthetic a source of danger in these cases, and that it was more dangerous than the incision. There was a possibility of spontaneous recovery without operation, and he had seen pathological appearances which seemed to show that this had occurred. The most important signs of perforation were some sudden change for the worse in the patient and a rise in the pulse rate. —Mr. C. A. Morton considered it was thoroughly worth while to operate in these cases, and that there was no definite *post-mortem* evidence that recovery after perforation was possible without operation.—Mr. Kyle, in replying, stated there had been neither collapse nor rise in the pulse rate in his case.

Mr. H. F. Mole read a paper on **two cases of ruptured tubal pregnancy.** *See* p. 39.—Mr. T. Carwardine mentioned a case in which there had been difficulty in diagnosing whether the condition present was due to ruptured tubal pregnancy or perforation of a gastric ulcer. He had had most difficulty in diagnosis in a case of torsion of the pedicle of a small ovarian cyst in association with pregnancy.

---

*February 11th, 1903.*

Mr. G. Munro Smith, President, in the Chair.

The meeting was devoted to the exhibition and discussion of cases.

Dr. E. Markham Skerritt showed a man of 62 with **anomaly of blood-vessels.** The man was troubled with shortness of breath, and for eighteen months his right arm had not been so useful for work as before, having become weaker. The right carotid near the root of the neck was much diminished in calibre. The radial and brachial arteries had very weak pulsation, and had thickened walls. A conspicuous abnormal artery passed from the second right intercostal space in front outwards over the chest to the axilla. Obviously the obstruction of vessels was of long duration. There was no evidence of aneurysm nor of embolism. The obstructed vessels appeared to be of normal size, but to have thickened walls, and probably this thickening accounted for the obstruction.—Dr. H. Waldo mentioned a case he had reported some time ago, which was in many respects similar to the above, and in which he had felt sure the obstruction was due to syphilitic endarteritis.

Dr. H. Waldo showed: (*a*) A case of **Lupus Vulgaris** of both sides of the face. The left side had been treated with the Finsen light for eight months. The other side of the face had improved as well as that under the special treatment. (*b*) A case of **Lupus Vulgaris** affecting the right side of the face. Under the Finsen light treatment a patch on the buttock and one on the left cheek had been rapidly cured. The right side of the face had also improved. (*c*) A case of Folliculitis.

Mr. A. W. Prichard showed a case of **simultaneous amputation of both breasts for carcinoma.** The patient was 24 years of age. At the time of her admission to the Bristol Royal Infirmary there was a hard swelling in the left breast beneath a scar. The scar was that of an operation performed nineteen months before, in which a tumour had been removed which was thought at the time to be benign, but which the microscope had subsequently shown to be malignant. The recurrent tumour was the size of a hen's egg, and somewhat adherent to the skin and to the pectoral muscle, and with a hard prolongation passing to the axilla. In the right breast there was also a hard tumour, the size of a pigeon's egg, and a separate swelling in the skin above. In each axilla hard glands could be felt. The patient made a rapid recovery after the simultaneous amputation of both breasts. It was now only a month since the operation, and Mr. Prichard proposed later to report the patient's further progress to the Society.

Dr. J. E. Shaw showed a case of **sclerodermia, with pigmentation and muscular atrophy.** Paræsthetic numbness had first appeared in the hands, about a year previously, followed by a similar affection of the legs. Afterwards much weakness developed of both arms and legs. Pigmentation had begun to appear five months ago. Sclerodermia affected the neck and face on the left side, and the upper and front part of the chest. There was marked wasting of the muscles of the arms. The rigidity of the skin of the palms prevented closure of the fingers. Pigmentation affected the hands and forearms and the lower part of the abdomen, but not the axilla nor the buccal mucous membrane. Though not markedly wasted, the legs were so feeble that the patient could not rise from the ground without assistance. Twelve days' treatment with thyroid extract had produced considerable improvement.—Dr. Watson Williams asked if the case might not be one of myxœdema, resembling sclerodermia.—Dr. Kenneth Wills alluded to the fact that the wasting of muscles was not confined to the area affected with sclerodermia. He had noticed that the patient was sweating well.

Mr. Paul Bush showed: (*a*) An infant, seven weeks old with a **cystic swelling in the right cheek,** which had been present since birth. The infant had been born without difficulty, and without the use of instruments. The swelling made the face asymmetrical. It was not a defined tumour, but a soft, elastic, somewhat diffuse thickening. It was translucent, and yielded clear fluid when explored with a needle and syringe. (*b*) A case of **obscure hip disease** in a youth of eighteen. Two years previously pain had been felt in the hip. He had been able to work all through the year 1902, and could still walk several miles, though he was a little lame. There was one inch of real shortening, which was due to alteration of the neck of the femur, not of the shaft. The movements were practically natural, except those of inversion and of abduction, which were diminished in range. The trochanter was elevated about three-quarters of an inch. A skiagram showed the angle of the neck of the femur was more acute than that of the other side (coxa-vara). (*c*) A case in which **the clavicle had been excised for sarcoma** in a girl of nineteen. The tumour had been noticed first, when quite small, eight months previously. It had steadily increased since, and had affected the middle third of the clavicle, extending four inches along the bone. The specimen was shown. (*d*) A case of **excision of the scapula for sarcoma** in a girl of sixteen. Two years previously pain had been first experienced. Three months later a swelling over the inner and upper part of the scapula had been noticed, and this had steadily increased. The inner two-thirds of the scapula were removed. There was a good deal of hemorrhage, and severe post-operative collapse. She had steadily improved since.—Mr. H. F. Mole considered that the tumour in the cheek could not be a dermoid, because it contained clear fluid.—Mr. T. Carwardine alluded to a paper he had read at the Pathological Society, in which he had attempted to show that cystic-hygromata of the neck were developed in relation with the branchial clefts from epiblastic inclusion, and he believed in the case shown that the cyst had developed in relation with the intermaxillary cleft in that way.—Mr. J. Lacy Firth said that the case of hip disease shown reminded him strongly of the case of double coxa-vara which he had shown to the society some years ago. The limitation of inversion and of abduction only were characteristic, and he had no doubt that the case was one of coxa-vara.

Dr. J. Michell Clarke showed: (*a*) **A case of Graves' disease.** The patient was a woman of 32. Ten months before she had been in the hospital for exophthalmic goître. Last Christmas she developed shooting pains in the legs and pains like that of cramp, with weakness, so that the legs gave way when walking. The exophthalmos was marked on the right side, slight on the left. On the left leg there was a local patch of hyperidrosis. The patch was pigmented, with hair upon it, and it flushed when secreting. (*b*) A case of **intracranial tumour** in a man of 40. Eighteen months ago, after exposure to the sun, occipital headache and vomiting came on. Similar attacks had gradually become more frequent, and for the last fourteen days had occurred every day. The patient had had five fits, with loss of consciousness since, and one well-marked epileptic fit on December 12th. Optic neuritis was present. (*c*) A case of **thoracic aneurysm with indirect signs.** For eighteen months there had been pain in the chest persisting on the right side. No pulsation could be discovered for many months. Last November pulsation and deficient resonance in the second right intercostal space developed, and there were deficient resonance, harsh breath-sounds, and occasional râles heard over the lower lobe of the right lung. The radial pulses were equal, and there was no tracheal tugging. (*d*) A case illustrating the **treatment of pancreatic diabetes by opium.** The patient was a boy of 16. He began to lose weight at 14. Last November he had been admitted to the hospital with weakness, drowsiness, and emaciation. In December he excreted about 12 ounces of sugar daily, and 108–116 ounces of urine. Diacetic acid and acetone were also present. He began to take liquor opii sedativus in the middle of the month, taking at first ♏x each day. Now he was taking ℥iss of tincture of opium daily, and excreting 48 to 60 ounces of urine with about 3 ounces of sugar, and had gained 10 lbs. in weight. None of the usual effects of opium were produced by the large doses given.

Mr. F. Richardson Cross showed: (*a*) Two patients for whom he had removed the lenses for high degrees of myopia. In one patient, as the result of the operation, vision had improved so much that, whereas before operation it had been $\frac{6}{24}$ with the use of $-20$ glass, now without a glass it was $\frac{6}{18}$. This patient was now anxious to have the other eye operated upon. In the second case vision before operation had been:

$$\text{R}\bar{c}\ -4\ \text{sph.} = \tfrac{6}{6}.$$
$$\text{L}\bar{c}\ -20\ \text{sph.} = \tfrac{6}{36}.$$

Now, after operation in the left eye, vision in that was $\frac{6}{9}$ without a glass. Mr. Cross emphasised the advantages these operations conferred on the patients. (*b*) A case in which severe **ectropion of the eyelids** had been greatly improved by the application of Thiersch skin grafts. The condition of the cornea had much improved.

Dr. A. Ogilvy exhibited a **case of ruptured choroid,** showing the entrance of one of the ciliary arteries through the sclerotic. The patient had fallen and struck the eye on a water tap. A large white patch was seen in the region of the macula with a vessel coming through the sclerotic visible in it. Round a portion of the wound the torn edge of the retina was visible, turned back on itself. The floor of the patch was depressed and showed a good deal of pigmentation. On admission there had been no perception of light, now it was $\frac{5}{24}$. Reasons were given for regarding the appearances to be due to rupture of the choroid rather than to congenital coloboma.—Mr. C. H. WALKER could not agree that the case was one of ruptured choroid. The appearances were exactly those of many cases of coloboma, and he thought the injury had occurred to an eye already affected with coloboma.—Mr. CROSS was also inclined to regard the case as one of macular coloboma.

Mr. C. H. WALKER showed a patient with (*a*) a **cyst of the iris.** There was a history of a wound with a chisel seven years previously. Dimness of sight had come on four or five weeks ago, and now vision was about $\frac{6}{18}$ in dull light. (*b*) A patient with **a strand of organised lymph in the anterior chamber** of the eye, the result of a wound from a broken wire nine years previously.

Mr. R. G. P. LANSDOWN showed: (*a*) A case of **amputation of the breast for carcinoma, with removal of rib.** At the operation the pleura had been opened and the lung had collapsed. Primary union without further complication had followed, and now, five months after the operation, the parts looked healthy. (*b*) A case of **atrophic scirrhus of the breast with secondary deposits.** Mr. Lansdown had advised operation four years ago, but it had been refused. The deposit in the left lower jaw was now half the size it had been, and a deposit in the pectoral muscle was undergoing atrophy also.

Dr. F. H. EDGEWORTH showed: (*a*) A boy of eighteen with **achondroplasia.** (*b*) A **case of acromegaly** in a woman. There was no evidence in the case of involvement of the optic chiasma. The patient had been shown two years before. The continuous administration of supra-renal and pituitary gland prevented the occurrence of pain in the arms, but it was doubtful if it had had any effect on the progress of the disease.

Mr. T. CARWARDINE showed a patient with **perityphlitic actinomycosis.** The patient had been successfully operated upon for appendicitis, and the wound healed up; but some weeks afterwards a swelling and two sinuses had gradually formed. From the discharges the ray fungus was obtainable.

Mr. H. F. MOLR showed: (*a*) A case of **Duchenne's birth palsy.** (*b*) A case of **injury of the testis.**

J. LACY FIRTH.

J. PAUL BUSH (*Hon. Sec.*).

